# Natural and Synthetic Repellents for Pest Management of the Storage Mite *Tyrophagus putrescentiae* (Schrank) (Sarcoptiformes: Acaridae)

**DOI:** 10.3390/insects12080711

**Published:** 2021-08-09

**Authors:** Naomi Manu, Mark Wesley Schilling, Thomas Wesley Phillips

**Affiliations:** 1 Department of Entomology, Kansas State University, Manhattan, KS 66506, USA; nmanu30@ksu.edu; 2 Department of Food Science Nutrition and Health Promotion, Mississippi State University, Mississippi State, MS 39762, USA; Schilling@foodscience.msstate.edu

**Keywords:** stored products, food additives, population growth, plant essential oils, terpenes

## Abstract

**Simple Summary:**

The ham mite is the major pest of dry-cured hams, aged cheeses, and specialty pet foods that are high in fats and proteins. Ham mites are also known to cause allergies in some cases for humans. The toxic fumigant gas methyl bromide had been used for years to control this mite pest, but it is being phased out of use due to its impact on the protective ozone layer of the earth’s upper atmosphere. Ham producers now require alternatives to methyl bromide for controlling mites. We conducted laboratory experiments with food-safe synthetic and plant-derived chemical repellents to keep mites away from dry cured hams. Our results showed that several of these repellents could effectively prevent ham mites from contacting and staying on treated pieces of ham, and that they would readily go to untreated ham pieces when given a choice. Further experiments found that mites would not feed on nor produce offspring when held on ham pieces coated with oils from thyme, lemon grass, rose, or a mixture of naturally occurring fat molecules. Our experiments suggest that these food-safe repellents might protect dry-cured hams from mites during their time in aging rooms by application to racks on which hams are aged or to the nets and packaging in which hams are held.

**Abstract:**

The fumigant pesticide methyl bromide (MB) was used for stored products, but it is now banned for most uses in many countries as an ozone-depleting substance. MB was the only pesticide used to manage the ham mite, *Tyrophagus putrescentiae*, which is the most significant pest of dry cured hams. Effective alternatives to MB are needed to develop integrated pest management (IPM) programs for this pest. This study evaluated plant essential oils and food-safe compounds as repellents to directly protect hams from infestation. Experiments to assess the repellency to orientation, oviposition, and population growth of mites on pieces of aged country hams were conducted. Test compounds at different concentrations were dissolved in respective solvents and compared to the solvent control. Results showed that C8910, a mixture of three short-chain fatty acids, and the sesquiterpene ketone nootkatone had repellency indices of (RI) of 85.6% and 82.3%, respectively, at a concentration of 0.1 mg/cm^2^, when applied to a Petri dish arena. DEET and icaridin were also tested but performed poorly with RIs below 70% even at 0.1 mg/cm^2^.The monoterpene alcohol geraniol had the highest RI of 96.3% at 0.04 mg/cm^2^. Ham pieces dipped in C8910 and nootkatone at 150 ppm each had RIs of 89.3% and 82.8%, respectively. In general, as the concentrations of test compounds increased, the numbers of eggs that were laid on these treated ham cubes decreased after the 48 h exposure time. Ham pieces dipped in different concentrations of test compounds and then inoculated with 20 adult mites showed a significant decrease in mite population growth compared to control pieces after 14 days. The results of these experiments suggest that some plant secondary metabolites and synthetic food-safe compounds could serve as potential alternatives for managing mites on hams.

## 1. Introduction

*Tyrophagus putrescentiae* (Schrank) (Acarina: Sarcoptiformes), known as the ham mite, cheese mite, or mold mite, is a cosmopolitan arthropod pest that infests a large number of durable stored foods and processed commodities with high-fat and high-protein content and moisture contents from 15 to 45% [1,2]. Ham mites usually live on the surfaces of high-value stored products such as dry-cured ham, other dried meats, cheeses, nuts, dried fruits, spices, cultured cheeses, and semi-moist pet foods, and they sometimes penetrate into the food to cause economic losses [3,4,5,6].

Southern dry cured ham is aged to achieve the desired taste, flavor, and texture, but mite infestation during aging can lead to regulatory action from meat inspectors [7]. Over the years, mite s have been problematic but effectively controlled solely by using methyl bromide, which served as a fast-acting and very effective fumigant [8]. However, methyl bromide is being phased out of use and is not available in most industrialized countries at this time because of its detrimental action to the ozone layer of the earth’s atmosphere [9].

The use of synthetic chemical pesticides to control insects and other arthropod pests is decreasing, since concerns have been raised based on human health, environmental effects, and pesticide resistance. Therefore, alternatives that are natural products and safe for both humans and the environment are being evaluated for pest control applications [10]. Consequently, a search into these natural products as alternatives to control insects and other arthropods can be useful. Safe natural products include essential oils, which are naturally occurring defensive compounds in plants, that include monoterpenes, sesquiterpenes, and diterpenes from plants that serve as protective allomones to repel and intoxicate phytophagous insect. Some essential oils are known for their acaricidal effects [11], which led us to investigate these compounds as repellents for ham mites.

Methods such as food-safe compounds incorporated in ham nets to prevent infestation of hams and traps to detect and monitor mite populations over space can serve as tools for integrated pest management for ham mites [12,13,14,15]. Abbar et al. (2016) tested different food-safe compounds such as propylene glycol (1, 2-propanediol), lard, ethoxyquin, and butylated hydroxytoluene in controlling experimental populations of ham mites after 14 days [12]. Recent work by Zhang et al. [15] demonstrated that 95% of ham mites oriented to ham cubes wrapped in untreated nets as compared to cubes wrapped with nets treated with food-safe repellents such as xanthan gum, carrageenan, propylene glycol alginate, and propylene glycol in a behavioral experiment at different concentrations.

This present study focuses on using terpenoids that occur naturally in many vascular plants as well as food-safe synthetic repellents to deter and mitigate ham mite populations for eventual use in commercial ham aging rooms. A fatty acid blend, C8910, is made up of three medium-chain fatty acids with C8, C9, and C10 normal chain lengths mixed in equal parts, which occur in some plants and were elucidated earlier as a safe pesticide [16]. Octanoic and decanoic acids are natural derivatives of palm kernel oil or coconut oil by the process of steam hydrolysis, while nonanoic acid is derived from tallow or from the action of lightening and ambient oxygen and oleic acid in the film of water droplets, which are less expensive and easy to access [17,18]. C8910 is used as both a repellent and an insecticide against susceptible and resistant strains of insects and other arthropods [19]. The commercial formulation of C8910 is repellent against biting and non-biting flies such as mosquitoes and acarids such as ticks [20]. Stractor, Inc. (Richmond, CA, USA) produces C8910, which is U.S. FDA approved and categorized as “generally recognized as safe” [18].

According to Mao et al. [21], nootkatone is a natural sesquiterpene that is obtained from grapefruit and has contact toxicity and repellency to some stored product insect pests such as the maize and rice weevils, *Sitophilus zeamais* and *S. oryzae* (Coleoptera: Curculionidae). Ticks, *Ixodes ricinus* (Linnaeus) (Ixodida: Ixodidae), have been effectively repelled by oxygenated compounds such as citronella, cinnamyl alcohol, geraniol, and α-pinene, which were isolated from the essential oil from flowers of the *Dianthus caryophyllum* and *Artemisia abrotanum* plant [22]. Carvacrol, geraniol, and citronella were found to repel *Anopheles gambiae* (Diptera) successfully at lower concentrations [23,24]. It was also discovered that the commercial synthetic insect repellent DEET (N, N-Diethyl-3-toluamide) is not only a broad-spectrum repellent but is very effective in repelling mosquitoes, since it is persistent in the skin of humans, unlike other synthetic compounds [25]. Citronella is a bio-pesticide that is safe for human use and has been approved by the United States Environmental Protection Agency since 1948 [26]. Citronella is used for protection against mosquito bites and prevents the invasion of stored food products by arthropod pests. Interestingly, citronella is more effective as a repellent than DEET in many applications [27,28,29,30]. Icaridin is another compound that has been evaluated for its ability to repel arthropod pests. One study suggested that Icaridin could be a very good substitute to DEET, since it repelled two species of ticks in a laboratory experiment [31]. We chose to include DEET and Icaridin in our studies as mite repellents because they are safe for humans, they are not natural products and thus are included as comparisons to natural products, and they have not been evaluated as repellents for stored product pests. These synthetics are considered food-safe and likely could be approved by food regulatory agencies to repel ham mites from hams and other foods in the future.

In order to ameliorate the problem of ham mites from dry-cured ham, the use of food-safe compounds which are registered to either repel or kill these pests is drawing research attention [12,15]. Therefore, the objective of this study was to assess the effectiveness of some food-safe compounds and essential oils as allomones for repelling ham mites. Additional experiments on the oviposition, infestation, and population growth of mites exposed to food-safe repellent-treated ham pieces were conducted using the most repellent compounds.

## 2. Materials and Methods

### 2.1. Mite Cultures and Ham Used

*Tyrophagus putrescentiae* (ham mites) were reared in glass jars (85 mm diameter, 160 mm height, volume of 917 mL) containing mite diet that were firmly sealed with labelled filter paper (Fisher Filter paper, diameter 7.0 cm, Pittsburgh, PA 15275, USA) in a metal lid ring. The diet was prepared as described in previous research projects in our laboratory [12,13]. The ingredients in the diet for rearing the mites included 5 g each of agar (ICN Biomedicals, Inc. Aurora, OH, USA); yeast (MP Biomedicals, LLC, Santa Ana, CA, USA); alphacel (ICN Biomedicals, Inc.); and mixed vitamins (Vanderzant modification vitamin mixture for insect diet; MP Biomedicals, LLC, Santa Ana, CA, USA). Other ingredients included 160 g of commercial dry dog food that was ground, and the liquids glycerol (Fisher BioReagents, Fisher Scientific, Pittsburgh, PA, USA), antifungal salt solution in ethanol (methyl-p-hydroxybenzoate, 15:85 g mL^−1^) (ICN Biomedicals, Inc.), and water in a ratio of 25:25:475 mL. These ingredients were mixed and brought to boil for 30 min. Then, approximately 30 mL of the cooked mixture was added to each jar that contained approximately 50 g of unground dog food kibble in the rearing jars. The jars were cooled to room temperature, and then mites were introduced to the new cultures. The cultures were firmly sealed and placed in plastic containers that were filled halfway up with soapy water to prevent the escape of mites. The plastic containers with the jars were placed in an incubator at 25 °C and 70% RH in total darkness. Hams used in this study were acquired as whole country hams (6–8 kg) that had been aged for a minimum of 3 months from a commercial dry cured ham company in Tennessee.

### 2.2. Chemicals

Table 1 lists the compounds studied and their suppliers.

### 2.3. Repellency of Test Compounds Using Two-Choice Orientation Assay

Two-choice behavioral tests were conducted by using glass Petri dishes (90 × 20 mm glass Petri dishes) as a behavioral assay arena to test the orientation of mites. Test compounds in acetone were on one side of the dish, and the other side contained only acetone as the untreated control. The floors of the glass Petri dishes were divided into two equal halves by drawing a line through the middle with a grease pencil. This also prevented the test compound solution from migrating to the control arena of the Petri dish before the solvent evaporated. Compounds used included the fatty acid blend of C8910, Icaridin, DEET, citronella oil, carvacrol, nootkatone, and geraniol. Application rates of 0.001, 0.02, 0.04, 0.06, 0.08, and 0.1 mg/cm^2^ were used for each test compound. Approximately 100 ham mites of mixed life stages were placed at the center of the Petri dish to determine which side the mites oriented toward. Mites were added by first placing a small rectangular piece of white paper with an area of 6 mm^2^ inside an active mite colony for 5 sec while being held with forceps. Mobile mites crawled onto the paper, and then, the paper was lifted out of the rearing jar and placed in the center of a given Petri dish floor. Mites were prevented from leaving the assay Petri dishes by applying a thin layer of vacuum grease along the inside upper 5 mm of the vertical sidewall of the dish. Once dishes were set up, repellency assays were conducted in a room with complete darkness at a temperature of 25 °C and 70% RH. Mites were allowed to move in the Petri dish for 30 min and then were counted based on the half of the arena they oriented to, which were either the treatment or the control.

### 2.4. Mite Orientation and Oviposition Bioassay

Initial experiments on mite repellency in Petri dish arenas (methods above) indicated that C8910, nootkatone, and geraniol were very effective at repelling mites and were examined further for how they may affect mite orientation to and oviposition on ham meat. C8910, nootkatone, and geraniol were evaluated in a similar behavioral assay using 90 × 20 mm glass Petri dishes (Pyrex^®^, Frankfurt, Germany) as arenas, each with a 90 mm diameter circular piece of black construction paper under the floor of each dish to allow for observation of the small white mites. Ham cubes of 5 × 5 × 5 mm (125 mm^3^) were used in two-choice assays. The ham cubes were cut from the same section of the whole dry-cured ham, with a new ham being used for each experiment. Three circles of 18 mm diameter were drawn with pencil on the black construction paper and centered along a line passing through the center of the paper, with one circle in the middle of the floor and one circle each at a distance of 5 mm from the side wall. Then, the circular construction paper was placed beneath the Petri dish and held in place with double-sided tape. Concentrations of 25, 50, 75, 100, and 150 ppm (µg/mL) of each test compound were made using acetone as the solvent. Treatment ham cubes were dipped in the various solutions, and the control cubes were dipped in acetone only, for 1 min, and then allowed to air dry for 60 min. For each of the five bioassay arenas for each treatment, one ham cube was treated with the test compound, and the control solvent-treated ham cubes were placed in each of the circles closest to the dish wall. Thirty adult mites of mixed sexes were released into the middle circle of each assay arena. Mites were prevented from leaving the bioassay dishes by applying a thin layer of vacuum grease along the inside upper 5 mm of the vertical wall of the Petri dish. The Petri dishes were covered and left on a bench in total darkness at 25 °C and 70% RH for 48 h without any disturbances until data collection. Mites that oriented to each of the ham cubes, which included those on the ham surface and on the floor within the circle outlining the ham cube were counted under red light in the dark room at 1, 2, 3, 24, and 48 h. Then, mite eggs laid by the end of this 48 h period were counted under a binocular dissecting microscope (200 × X) and recorded.

### 2.5. Mite Population Growth

The population growth of mites on ham that was treated with test compounds at different concentrations was determined. The food-safe compound, C8910, and the essential oil terpenoids, citronella, carvacrol, geraniol, and nootkatone were evaluated in separate experiments using a completely randomized design of five replicates per concentration (treatment) of a test compound as done previously [12].

Dry-cured ham cubes (25 × 25 × 25 mm) were dipped in a given concentration of the test compound; water or acetone alone (depending on the compound) were used as controls. Concentrations of 1% and 10% of test compound were compared to the solvent control. Five ham cubes were placed in 200 mL of the test solution in a 500 mL beaker for 1 min, removed and left on filter paper in an open 90 cm glass Petri dish to dry for 60 min. Then, treated ham cubes were placed separately inside small glass Mason jars (216 mL, 65 mm diameter, 55 mm height; Ball Corp., Broomfield, CO, USA), and 20 adult mites of mixed sexes were introduced for infestation; a similar procedure was followed for the controls. Ham mites were prevented from leaving the assay jars by applying a thin layer of vacuum grease along the inside upper side wall 15 mm from the bottom. The jars were covered with their screw-on ring lid with a circular piece of filter paper for ventilation (in place of the metal lid intended for such jars); then, they were placed in opened plastic shoe boxes filled half-way with soapy water to prevent any escaping mites from contaminating the laboratory. Incubation was done for 14 days at 25 °C and 70% RH with 16:8 h L:D photoperiods. Live mites and progeny (mobile mites of adults and immatures) in each jar were counted using a microscope (as above).

### 2.6. Statistical Analyses

Repellency index (RI) for the initial two-choice orientation bioassays was calculated using:

RI = ((Nc − Nt)/(Nc + Nt)) × 100, whereby Nc = the number of mites on the control side and Nt = the number of mites on the treated side [32]. Data were statistically analyzed using SAS software 9.4 (version 2012). We subjected the RI proportional data from behavioral assays using the arcsine transformation prior to statistical analyses. We used one-way ANOVA to determine the significant effects of the treatment concentrations on mite orientation. Differences between the treatment and control were determined with the Ryan–Einot–Gabriel–Welsch and Quiot (REGWQ) test for multiple comparisons at a probability of *p* < 0.05. The residency of mites on ham cubes with different coatings and within the circles were subjected to a two-way ANOVA for each observation time at different concentrations. Mean RIs at different observation times over 48 h and for concentrations of given compounds were separated by the honestly significant difference Tukey–Kramer test when the F-test of the ANOVA was significant at *p* < 0.05. With the oviposition study, statistical analyses for differences between treatment and controls for the percentage of eggs laid on each were determined with the Student’s *t*-test following arcsine transformation. For the population growth experiment, data were analyzed with SAS for significant effects of test compound concentration on mite numbers. Then, the REGWQ test was used to separate population growth means for multiple comparisons at a probability of *p* < 0.05. We used SigmaPlot Version 11 to draw graphs for results as needed after the data were analyzed.

## 3. Results

### 3.1. Two-Choice Orientation Assays

**Figure 1:**
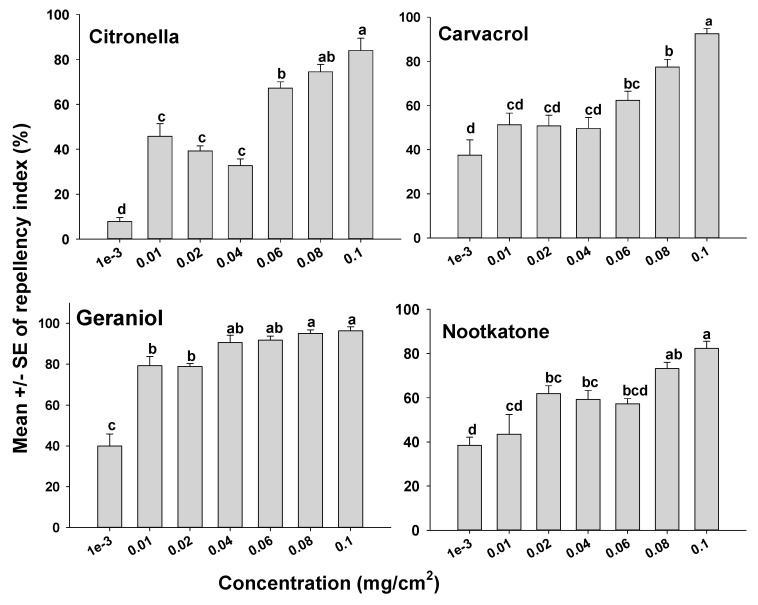
Orientation response of *T. putrescentiae* on glass surfaces treated with terpenes in a laboratory two-choice behavioral assay. ANOVA determined that there was significant variation among concentrations for each of the terpenes tested (F-value range 1.27–49.92; *p*-value < 0.001). Mean RIs (± SE; *n* = 5) for a given terpene with the same letter are not significantly different (REGWQ; *p* > 0.05).

**Figure 2:**
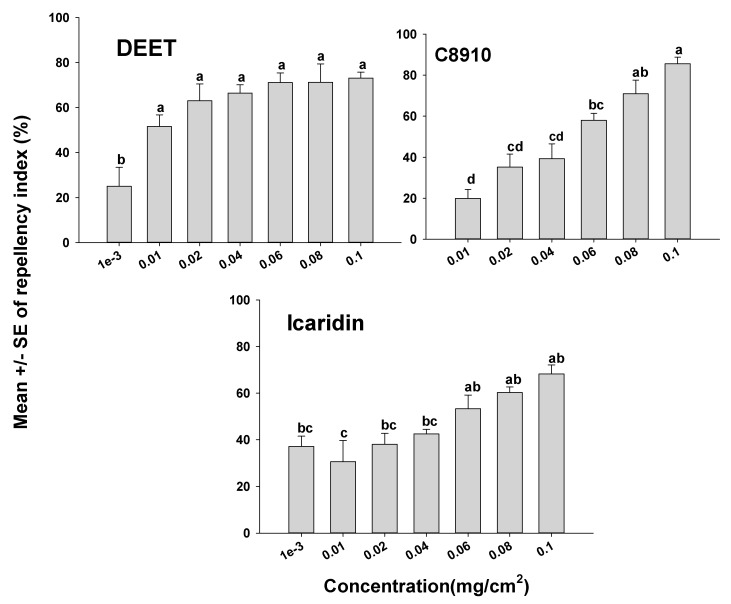
Orientation response of *T. putrescentiae* on glass surfaces treated with synthetic repellents in a laboratory two-choice behavioral assay. ANOVA determined that there was significant variation among concentrations for each of the compounds tested (F-value ranged from 6.12 to 19.25 and *p*-value ranging from <0.01 to 0.03). Mean (± SE; *n* = 5) RIs for a given repellent with the same letter are not significantly different (REGWQ; *p* > 0.05).

The orientation assays demonstrated that mites oriented more toward the untreated side of the Petri dishes than to the treated side as indicated by positive RIs in all cases. However, depending on the compound tested, some compounds appeared more repellent than others (Figure 1 and Figure 2). It was also apparent that the concentration of a compound used had an effect on mite orientation, since as the chemical concentration increased, more mites moved away from the treated side, representing a concentration-dependent response. With the terpenoids on the glass surfaces, geraniol had a high RI of 88.9% at a concentration of 0.04 mg/cm^2^, which was not different (*p* > 0.05) from geraniol at 0.1 mg/cm^2^ with an RI of 96.3%. The repellency of the other terpenoids at concentrations of 0.08 and 0.1 mg/cm^2^ for carvacrol, citronella, and nootkatone with RIs of 77.5% and 92.5%; 74.5% and 84.0%; and 73.2% and 82.3%, respectively (Figure 1). The synthetic compounds C8910, DEET, and icaridin were less repellent than the natural products, with the highest repellency indices at 0.1 mg/cm^2^. The three synthetic compounds C8910, DEET and icaridin had mean RIs of 85.6, 73.1, and 68.2%, respectively at 0.1 mg/cm^2^, which were lower than the RIs of the essential oils (Figure 2).

### 3.2. Mite Orientation and Oviposition on Treated Ham Cubes

Similar to results from the dish arena assays, the orientation of mites to ham pieces was less as the concentration of a given repellent increased (*p* < 0.05), regardless of exposure time (Table 2). All three compounds were repellent to some degree at all concentrations, and many of them showed no significant differences of RI over the five different time periods of the assays.

With regard to the number of eggs laid within the period of 48 h, the raw data showed a range of 1–50 eggs for the solvent-treated controls and 0–27 eggs for the treated ham cubes. Nootkatone-treated cubes of ham had the least number of eggs with a range of 0–4, followed by geraniol with 0–7 and C8910 had 1–27 eggs, respectively. The proportion of eggs laid on untreated ham cubes was always significantly higher than the proportion laid on untreated cubes (Table 3). There were no eggs, to very few, laid on ham pieces treated with either nootkatone or geraniol for any of the five concentrations tested. Therefore, these two terpenes could serve as good repellents to prevent the infestation of dried ham. 

### 3.3. Mite Reproduction Assay

**Figure 3:**
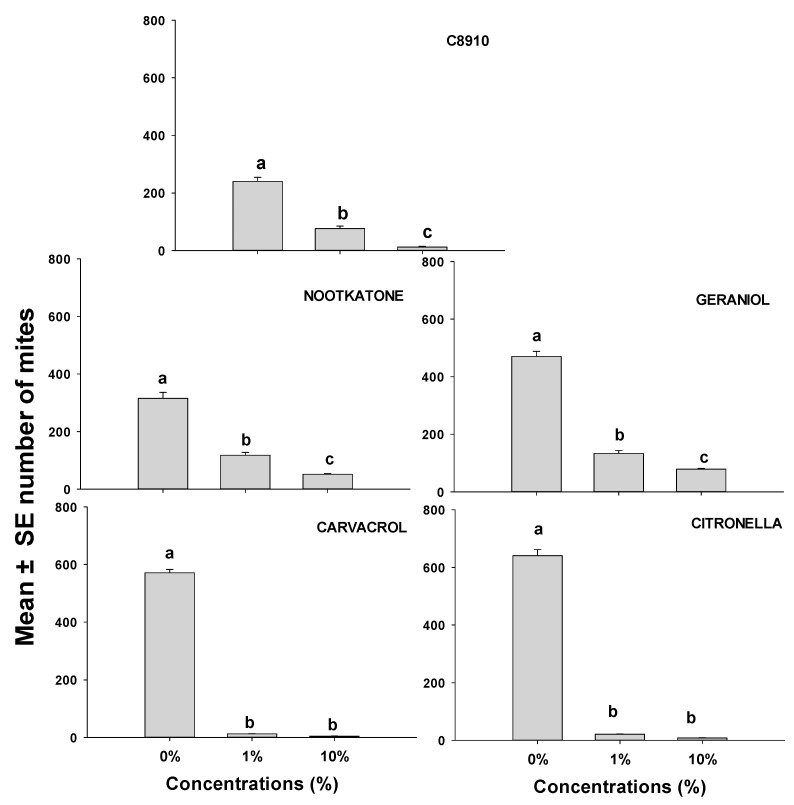
Mean (± SE; *n* = 5) number of mobile *T. putrescentiae* progeny 14 days after inoculation of 20 mites on small dry-cured ham cubes treated with either solvent only or one of five different repellents at two concentrations. Means with the same letter are not significantly different at *p* > 0.05 using REGWQ and with ANOVA F-values of 7.29–12.16; *p* < 0.01.

This study focused on the population growth of ham mites on ham cubes when they were treated with either C8910, nootkatone, geraniol, citronella, or cavacrol, all of which were effective at repelling mites (Figure 1 and Figure 2; Table 2 and Table 3). Mite numbers produced on ham cubes dipped in solutions of C8910 and the four terpenoids were significantly lower in all treatments than the number of mites produced on the solvent controls (Figure 3). The terpenoids at different concentrations caused a reduction in mite reproduction after 14 days. Mite numbers on ham cubes treated with either a 10% concentration of nootkatone, geraniol, C8910, citronella, or carvacrol in acetone were significantly reduced to means (and SEs) of 51.6 (3.67), 78.8 (2.99), 12.4 (4.06), 8.2 (1.43), and 4.4 (0.74) respectively, when compared to controls. There were no significant differences observed between 1% and 10% concentrations in citronella and carvacrol, whereas the other oils differed significantly at these concentrations. This makes citronella and carvacrol the most effective at reducing the population of mites when used at dosages of 1 or 10%. Generally, all essential oils and the fatty acid mixtures reduced mite population growth substantially when applied on ham pieces.

## 4. Discussion

This research provides evidence that some naturally occurring plant compounds and other common synthetic repellents will repel and reduce the population growth of ham mites when applied at various concentrations in the laboratory. In recent times, a common way to study the behavior and ecology of animals is by choice bioassays [33]. Choice bioassays were employed for the study of ham mite behavior in this research. The results of the relationship between ham mites and the different food-safe compounds demonstrated that ham mites readily moved away in the presence of these test compounds. In the behavioral bioassays, the different essential oils had strong repellency against *T. putrescentiae* on glass surfaces. Ham mites oriented to the control side of the Petri dishes anytime they were allowed to stay undisturbed on the bench for 30 min. The few that managed to cross the line that separated the control and treated sides retracted themselves quickly to avoid the treated side when viewed under the microscope. The ability of essential oils used in these studies to repel other stored product insect pests from commodities has been widely studied e.g., [34,35,36]. Not only are essential oils used to repel insects from food commodities, they are also used for health purposes to prevent malaria [37], since they repel mosquitoes from biting humans [38].

The essential oil geraniol was the most effective compound for repelling mites of those tested, with an RI of 96.3%, which indicates that geraniol has potential for application in the management of ham mites. Past studies confirm that geraniol strongly repels both red flour beetles and booklice in comparison to citronellol and limonene when applied to control these stored product insect pests [15,39]. Another study found that mechanical diffusers with geraniol had a 97% repellent rate compared to other essential oils such as citronella and linalool diffused for repelling mosquitoes [38]. There is substantial information of geraniol having a higher tendency to repel arthropods than other essential oils [40]. The results from our work also indicate that geraniol is more repellent against ham mites than other essential oils tested, which is similar to what has been reported for mosquitoes by Hao et al. [41].

Citronella and carvacrol have the potential to effectively repel mites at higher concentrations. Citronella has been used in food packaging to protect commodities from insect pests. Citronella protected treated food-containing cartons for 2-week periods and minimized the infestation of insects by 50% at concentrations as low as 0.2 g/m^2^ of carton board [42]. Likewise, citronella incorporated in food packages was found to effectively repel the red flour beetle, *Tribolium castaneum*. [43]. The results described here indicate an RI of 84.0% when ham mites were exposed to a glass surface treated with citronella oil at a concentration of 0.1 mg/cm^2^.

Carvacrol is mostly known for its toxicity against arthropod pests more so than its repellency potential; however, when used in small concentrations, it can protect commodities by repelling arthropod pests [44]. The results from our study suggested a significantly higher repellency index of 92.5% at a concentration of 0.1 mg/cm^2^, which indicates potential for use as a repellent to manage mites. In one study [44], carvacrol was the best repellent of all tested compounds against ticks and mosquitoes. Although carvacrol is known to be effective, it easily loses its potential of repelling insect pests after 7 days [45].

Nootkatone was less effective at repelling mites when compared to the other terpenes that were tested. However, due to nootkatone’s mild odor in comparison to other essential oils, it is recommended for consideration in further experiments, since it protected ham pieces from ham mites by reducing the number of eggs laid by the mites. Earlier work with nootkatone reported repellency percentages that ranged from 46.3 to 93.1% and 39.2 to 67.3% against maize and rice weevils, respectively, as nootkatone concentrations increased from 0.05 to 5% (*w*/*v*) in methanol [21]. It was concluded that nootkatone was a good repellent candidate for insect pest management. Similarly, when nootkatone was used in the present study, the highest repellency index was achieved at the highest concentration of 0.1 mg/cm with an RI of 82.3%. 

The U.S. Environmental Protection Agency (EPA)-approved repellents that are used to repel disease-causing arthropods such as flies, ticks, and mosquitoes were evaluated here for their efficacy at controlling mites. Icaridin and DEET are well-known repellents for application to human skin that have the potential to repel biting insects and arachnid pests such as mosquitoes and ticks. According to our data, C8910, which is EPA-approved as a fly repellent on livestock, significantly repelled ham mites at higher concentrations, while DEET performed poorly as a mite repellent. C8910 is commercially marketed for use on humans and livestock in southern regions of Africa to control biting flies [16]. The present study indicated that the repellents DEET and icaridin were the least effective at repelling ham mites compared to all the other compounds tested (Figure 2). The exact mode of action for biting insects and ticks to not contact or move away from synthetic repellents is not understood, although there is evidence that repellents work through interactions with odorant receptors and gustatory receptors in arthropods [46].

Our orientation and oviposition experiment found that mites avoid ham cubes treated with C8910, nootkatone, and geraniol, and they were then drawn to oviposit on the untreated ham pieces (Table 3). These three compounds lacked efficacy at repelling mites at the lower concentrations of 25 and 50 ppm. Recent work suggests that the quality of the plant material from which an essential oil is obtained and its composition of volatile natural products may be different than that of the purified essential oil compounds [47]. This was evident in our results from the geraniol and nootkatone-treated ham cubes. Pieces of ham were available to the mites on either side of our test arenas (treated or untreated) in this study, but it was clear that ham mites tended to stay on the untreated ham, and we observed that the adult mites were not dying when near high concentrations of these compounds for up to 48 h. It was clear that the treated ham affected the number of eggs laid on untreated ham cubes for up to two days after treatment (Table 3). This choice for oviposition on untreated ham cubes was the same as in our previous work [12]. Mites were apparently repelled by the smell of the treatment compounds and thereby settled on the untreated ham for oviposition on the opposite side of the Petri dish. Repellency and negative orientation is probably adaptive because nymphs from any eggs laid on or near ham pieces treated with repellents may fail to eclose or the nymphs might die soon after eclosion. The activity of these volatile mixtures of natural products is dependent mainly on the type and quality of plant material that the oil is derived from and the process by which the manufacturer extracted the product [48]. With respect to C8910, the blend of synthetic short-chain fatty acids, there was relatively good reduction in the fecundity when pieces of ham were treated with it. Aside from these short chain fatty acids, other fatty acids have been studied previously. Caproic (six carbons) and propionic (three carbons) acids at 1 and 2% (by weight) caused a 100% reduction in the oviposition of *T. putrescentiae* from ham pieces [49]. However, these compounds applied to treated ham nets gave netted ham slices a moderate difference from untreated controls for a human taste panel. However, the C8910 had potential for achieving better consumer acceptability when mixed with other food-safe oils [50].

There has been very little work done to investigate the repellency of these food-safe plant-derived compounds against *T. putrescentiae*, although a lot of work has been conducted on the efficacy of these compounds against several species of stored product insect pests [10,37]. The most effective essential oils at repelling mites were further investigated to assess their acaricidal effect on mite populations on ham. Results with terpenes as mite repellents are in agreement with earlier work on insects [51,52] that reported results of higher toxicity of citronella and carvacrol against insect pests. Similar results were found when these compounds were used in controlling insect pests of stored grains [37].

In the population growth study, the results indicated that C8910, citronella, and carvacrol prevented the growth of ham mite populations over 14 days of storage. The low number of ham mites on treated ham cubes at 14 days suggests that mites may have been inhibited from laying eggs on the treated ham, hence preventing population growth from the initial number of 20 mites inoculated on the ham pieces at a concentration of 10% in the dip. Similar results were obtained in Abbar et al. [12] when ham cubes were dipped in other safe and approved food additives, which suggests that the application of various food-safe compounds may be an effective way to prevent infestation by ham mites on dry-cured hams. Abbar et al. tested compounds not known to be directly toxic to mites and insects, so the hypothesis of reduced oviposition leading to reduced population growth may be valid. However, in our work using the same population growth assay, it is possible that the low population on treated ham cubes resulted from the mortality of some or all of the initial 20 mites in a jar caused by the toxic volatiles of these repellents. This possibility requires further research. A recent study from our group showed that the addition of C8910 to nets used for covering and hanging country hams during aging clearly had reduced mite numbers on ham compared to ham in nets with no C8910 [50]. Although C8910 had better efficacy at inhibiting *T. putrescentiae* population growth in the present work, its strong smell after application needs further study for consumer suitability. Likewise, hams aged in nets containing the essential oils found effective at repelling mites in the current study would need to be evaluated for acceptability by human taste panels. Instead of the use of safe repellents such as those studied here in nets used to hang and age hams, it may be effective to coat non-food structural surfaces (e.g., floors, walls, hanging racks, etc.) within an aging room to prevent mites from crawling onto aging hams for a distance of several meters. Future research should be done at both laboratory scale and commercial scale to determine if these natural and synthetic repellents can keep mites away from hams on aging racks. The use of safe repellents could be a viable preventive component of IPM for high-value dry-cured hams.

## 5. Conclusions

The use of food-safe compounds such as terpenes from plant essential oils and a mixture of synthetic short-chain fatty acids might provide a reasonable way to effectively prevent the infestation of ham mites on dry-cured hams during aging. Food-safe compounds can be incorporated into integrated pest management programs as a preventive measure to control ham mite infestations, since methyl bromide fumigation is being phased out of use. Our study confirms the potential of these compounds to serve as repellents against ham mites on dry-cured ham.

## Figures and Tables

**Table 1:** Plant-derived terpenes and synthetic repellents that were evaluated for their efficacy at repelling mites and suppressing mite population growth.

Category	Commercial Compound Name	Chemical Name or Natural Source	Supplier ^2^
**Plant Terpenes**	Carvacrol	Thyme, *Thymus* spp.	Sigma Aldrich
	Citronella oil ^1^	Lemon grass, *Cymbopogon* spp.	Sigma Aldrich
	Geraniol	Rose oil, *Rosa* spp.	Sigma Aldrich
	Nootkatone	Grapefruit, *Citrus sinensis* × *C. maxima*	Sigma Aldrich
**Synthetic repellents**	C8910	Octanoic, Nonanoic and Decanoic acids	Emery Oleochemical LLC
	DEET	N, N-Diethyl-3-methylbenzamide	Sigma Aldrich
	Icaridin	1-(1-Methylpropoxycarbonyl)-2-(2-hydroxyethyl)piperidine	Sigma Aldrich

^1^ Terpenes that make up citronella oil are as follows: citronellal (32–45%), geraniol (21–24%), geranyl acetate (3–8%), citronellol (11–15%), and limonene (1–4%). ^2^ Sigma Aldrich is located in St. Louis, MO, USA and Emery Oleochemical LLC is located in Cincinnati, OH, USA.

**Table 2:** Orientation of *T. putrescentiae* to dry-cured ham pieces coated with three different repellents vs. a water-control in laboratory two-choice bioassays. Mean repellency index, RI, (± SE; *n* = 5) of mites orienting over time to ham pieces treated with repellent solutions of different concentrations (ppm).

Compound	Time (h)	Concentration (ppm)				
		25	50	75	100	150
**C8910**	**1**	20.0 (7.3) Bc	52.0 (9.8) Aab	38.7 (2.5) bc	68.0 (2.5) a	69.3 (4.5) a
	**2**	38.7 (4.9) ABb	66.7 (6.7) Aa	62.7 (3.4) a	73.3 (6.3) a	74.7 (5.3) a
	**3**	60.0 (3.7) Ab	74.7 (4.4) Aab	65.3 (6.5) ab	82.7 (2.7) a	74.7 (4.9) a
	**24**	26.7 (8.7) Bc	70.7 (12.8) Aab	41.3 (9.3) bc	60.0 (8.7) abc	89.3 (4.9) a
	**48**	28.6 (12.4) Bbc	13.2 (3.6) Bc	73.3 (11.4) a	53.7 (12.1) ab	87.0 (6.3) a
**Nookatone**	**1**	36.0 (5.4) Ba	50.7 (6.2) a	61.3 (7.7) a	68.0 (11.4) a	68.0 (9.0) a
	**2**	44.0 (6.5) ABc	58.7 (8.3) bc	62.7 (7.8) bc	86.7 (6.3) a	77.3 (3.4) ab
	**3**	42.7 (5.8) ABb	52.0 (6.5) ab	65.3 (6.8) ab	76.0 (7.5) a	78.7 (7.7) a
	**24**	45.3 (9.0) ABb	47.3 (12.8) b	48.0 (10.6) b	50.7 (17.8) b	82.8 (5.4) a
	**48**	56.3 (8.6) Ab	55.9 (13.5) b	57.4 (10.5) b	85.3 (3.9) ab	90.2 (4.8) a
**Geraniol**	**1**	62.7 (8.6) a	66.4 (8.9) a	71.8 (7.5) Aa	75.5 (2.6) a	84.7 (7.4) a
	**2**	45.2 (9.7) b	63.8 (7.6) ab	60.2 (7.8) ABab	70.5 (8.8) ab	82.3 (8.9) a
	**3**	57.4 (5.7) b	71.8 (9.1) ab	62.7 (8.0) ABab	70.4 (6.1) ab	89.5 (4.5) a
	**24**	49.2 (7.7) b	40.1 (6.1) b	33.1 (6.9) Bb	59.1 (11.6) ab	73.0 (4.4) a
	**48**	58.5 (5.8) a	66.6 (5.3) a	64.1 (8.7) Aa	81.5 (5.7) a	83.3 (7.6) a

Means followed by the same letter are not significantly different (*p* > 0.05). Lower case letters indicate significant differences among mean RIs at different concentrations in a row, while upper case letters indicate significant differences among RIs for each exposure time in a column for a given concentration. Exposure times with no upper-case letters did not have significant differences among RIs.

**Table 3:** Mean proportions of eggs laid by *T. putrescentiae* (± SE; *n* = 5) on small dry-cured ham pieces treated with various food-safe compounds compared to untreated ham pieces after 48 h in a laboratory two-choice behavior bioassay. Student’s *t*-tests following arcsine transformations of proportions were performed.

Compound	Concentration (ppm)	Mean (± SE)	Number of	Eggs @ 48 h	
C8910		Control	Treated	*t*-Value (df)	*p*-Value
	25	64.7 (2.9)	35.3 (3.0)	7.0 (4)	<0.01
	50	62.2 (4.6)	37.8 (4.6)	3.6 (4)	<0.01
	75	90.2 (3.6)	9.8 (3.5)	15.8 (4)	<0.01
	100	78.9 (6.7)	21.1 (6.7)	6.1 (4)	<0.01
	150	90.9 (4.8)	9.1 (4.8)	12.1 (4)	<0.01
**Nootkatone**	25	76.7 (19.4)	3.3 (3.3)	3.7(4)	<0.02
	50	92.0 (8.0)	8.0 (8.0)	7.4 (4)	<0.01
	75	81.7 (8.9)	18.3 (8.9)	5.0 (4)	<0.01
	100	100.0 (0.0)	0.0 (0.0)	-	-
	150	98.2(1.82)	1.8 (1.8)	37.5 (4)	<0.01
**Geraniol**	25	92.5 (7.5)	7.5 (7.5)	8.0 (4)	<0.01
	50	96.7 (3.3)	3.3 (3.3)	19.8 (4)	<0.01
	75	91.0 (5.6)	9.0 (5.6)	10.4 (4)	<0.01
	100	96.7 (3.3)	3.3(3.3)	19.8 (4)	<0.01
	150	100.0 (0.0)	0.0 (0.0)	-	-
